# Minimizing quay crane downtime in container terminals using genetic algorithms with a case study of Tangier MED Port

**DOI:** 10.1038/s41598-025-29190-0

**Published:** 2025-11-23

**Authors:** Hamza Garmouch, Otman Abdoun, Oumaima Garmouch

**Affiliations:** 1 Present Address: ISISA Team, Faculty of Science, Abdelmalek Essaadi University Tetouan, Tetouan, Morocco; 2 SMPNT, Faculty of Science, Abdelmaled Essaadi University, Tetouan, Morocco

**Keywords:** Downtime, Automated container terminals (ACT), Metaheuristic methods, Genetic algorithm (GA), Particle swarm optimization (PSO), Simulated annealing (SA), Engineering, Mathematics and computing

## Abstract

The problem of quay crane downtime continues to challenge container terminals across the globe, particularly in fully and partially automated ones like Tangier MED Port in Morocco. The persistent breakdowns of the cranes increase vessel turnaround times, delay completion times, add to the cost burden, diminish terminal productivity. This study formulates a genetic algorithm (GA) model aimed at reducing quay crane downtime due to planned maintenance, unplanned failures, equipment idle time coordination, and job scheduling performed within a single optimization framework. Unlike previous methods that address individual aspects in isolation, our approach models all crane operations to optimally reduce idle time and disruptions. The simulation results substantiate the claims that the proposed GA significantly improves crane and terminal efficiency. Also, analysis done in comparison to PSO and SA showed that GA is capable of providing better and more scalable solutions in modern container terminals. Specifically, GA achieved an average downtime of 98.3 min, compared to 99.5 min for PSO and 197.8 min for SA, confirming its superior performance.

**Supplementary Information:**

The online version contains supplementary material available at 10.1038/s41598-025-29190-0.

## Introduction

Container terminals play a critical role in the global logistics chain, serving as the primary interface between maritime transport and inland distribution networks. They facilitate the rapid and efficient transfer of goods from ship to shore, significantly affecting the overall performance of international trade operations. Among the equipment used, quay cranes are particularly central to terminal operations. Since they enable the direct handling of containers between vessels and the quay, quay cranes are widely regarded as the most critical asset in any container terminal ^1^. Their efficiency directly influences port throughput, service time, and cost-effectiveness ^2,3^. However, quay cranes are frequently affected by various disruptions, including mechanical failures, adverse weather conditions, and system or maintenance issues. These problems are even more pronounced in automated and semi-automated container terminals, where system complexity can amplify the impact of downtime ^4,5^. Such issues result in increased vessel waiting times, prolonged turnaround cycles, and delays in land-side operations, ultimately leading to reduced terminal productivity and profitability ^6^. Operational inefficiencies in quay crane usage pose a major concern for maritime logistics a sector that supports global commerce and economic development. This is particularly important for strategically located countries like Morocco, where maritime trade serves as a lifeline for economic expansion. Due to its geographic location between Europe and Africa, Morocco plays a pivotal role in international trade. Ports such as Tangier MED have evolved into major transshipment hubs, streamlining the movement of goods between trucks, trains, and vessels ^7^. These ports function as critical logistics platforms, supplying global supply chains and boosting the competitiveness of national industries ^8^. Despite the strategic importance of container terminals, they still face numerous operational challenges. Among the most prominent are excessive Makespan and long vessel port stays ^9,10^. Makespan, defined as the total time required to complete all container handling tasks, directly affects terminal throughput and ship turnaround ^11^. Port stay the time a vessel remains at berth adds to shipping costs and can disrupt tightly scheduled supply chains ^12^. Reducing quay crane downtime is one of the most effective strategies to improve both metrics and thereby enhance overall terminal performance. Several studies have employed optimization techniques to tackle related problems in terminal operations, such as berth scheduling, yard planning, and equipment allocation. These include both exact mathematical models and metaheuristic algorithms like Genetic Algorithms (GA), Particle Swarm Optimization (PSO), and Simulated Annealing (SA) ^13^. While these approaches have shown success, most focus on isolated components rather than integrated solutions. Very few studies explicitly address quay crane downtime in a holistic manner that includes both planned maintenance and unplanned disruptions, along with synchronized scheduling of jobs and equipment. This paper aims to fill that gap by developing a Genetic Algorithm-based model for the optimization of quay crane operations, specifically targeting downtime reduction in an automated container terminal context. The proposed model integrates multiple real-world operational variables planned maintenance windows, random failures, resource coordination, and job prioritization into a single optimization framework. The primary goal is to reduce idle time and improve crane utilization, ultimately increasing terminal throughput and efficiency. The remainder of this paper is structured as follows: Section "Review of optimization techniques in container terminals" provides a literature review covering recent optimization methods in port operations. Section "Problem statement and mathematical formulation" presents the problem definition and mathematical formulation. Section "Proposed genetic algorithm approach" details the implementation of the Genetic Algorithm. Section "Evaluation of scalability and sensitivity" offers a comparative analysis with other metaheuristics, and Sect. "Performance analysis and comparative study" concludes with key findings and directions for future research.

## Review of optimization techniques in container terminals

Optimizing quay crane operation is very important in automated container terminals to improve the efficiency of the whole port and reduce operational costs. Several research works have been conducted in this area, focusing on scheduling, resource allocation, and optimization of downtime issues. This section reviews some existing studies, classifying them according to the key themes: optimization techniques, integrated scheduling and resource allocation, and automation challenges. This review has revealed some gaps in the ongoing approaches and then positioned the proposed GA as a new contribution to the state of the art. different optimization methods have been proposed to tackle quay crane scheduling and maintenance challenges, ranging from exact solutions to heuristic and metaheuristic approaches. Genetic algorithms (GAs) have been widely adopted for their ability to efficiently explore large solution spaces and handle complex interdependencies. The makespan problem in ACT has been the subject of numerous studies over the past ten years. In light of the uncertainty associated with the discharge process, a study by Abyaneh et al. ^14^ has compiled various issues pertaining to integrated scheduling for different disparate equipment and maintenance activities in a container terminal. In order to manage operations across the three primary container terminal areas quay, storage, and clearance the authors created a novel optimization model. This model solely concentrates on gantry crane maintenance; it includes straddle carriers, trucks, trailers, and gantry cranes. Using machine learning and robust optimization approaches, a hybrid two-stage solution was given to realize the impact of maintenance uncertainty. The results showed that the suggested approach outperformed all other scheduling approaches currently in use, cutting down on downtime and improving transportation efficiency. The research makes a significant contribution to the area by addressing a problem that had previously been mainly ignored by integrating maintenance programming into container terminal scheduling. This kind of scalability offers a useful method for managing uncertainty. Evolutionary approaches like Genetic Programming (GP) have also shown promise in terminal logistics problems. For instance, Đurasević and Đumić ^15^ applied GP to automate decision-making in the container relocation problem (CRP), generating efficient relocation rules that outperformed manually designed ones. While CRP differs from quay crane downtime, their work highlights the adaptability of evolutionary algorithms to complex scheduling challenges in container terminals. Wang et al. present the berth allocation and quay crane scheduling problem under robust optimization with integrated and uncertain conditions ^16^. The authors note that handling times are normally disrupted at container terminals. thus, an approach is needed that could ensure reliability in uncertain conditions. The model of optimization put forward aims to reduce delays while being feasible in dynamic scenarios. These directions, in numerical experiments with real data provided by a large international port, have demonstrated that it can improve schedule reliability. However, the authors identify considerable computational cost increases for larger sets as one important bottleneck to scalability regarding real-time applications. Strong optimisation methods can actually cope with uncertainties in terminal operations; however, this usually has to come along with some trade-off in computational efficiency, as stated in this research. The authors indicate that hybrid optimization methods or heuristic approaches may be developed to reduce computation time for large instances, with minimal compromise on solution quality. This research provides useful insights into improving the reliability of port operations in disrupted environments, but it has left the integration of maintenance scheduling and its interdependencies with automated equipment unaddressed. The article by Li and Hu presents a preventive maintenance scheduling model for quay cranes in container terminals ^17^. The authors emphasize that while planned maintenance is essential for minimizing unexpected equipment failures, it often leads to significant operational downtime. Their mixed-integer linear programming model aims to optimize maintenance intervals, thereby reducing the adverse effects on terminal operations. Applied to both small and medium-sized datasets, the model demonstrated an average reduction in downtime of approximately 15% compared to existing methods, highlighting its robustness in maintenance scheduling. However, the study does not account for operational constraints arising from the interdependencies of equipment such as Automated Guided Vehicles (AGVs) and Automated Stacking Cranes (ASCs). Additionally, the model’s scalability to larger, more automated terminals remains unexplored, leaving its effectiveness in such environments uncertain. The authors suggest that incorporating real-time adaptability and integration with other terminal operations could enhance the model’s applicability in dynamic settings. Rahman et al. have presented a hybrid genetic algorithm, (QCDC-DR-GA), to optimize the container loading/unloading operations by combining quay crane dual-cycling with dockyard rehandle minimization ^18^. They indicate that usually in the traditional optimization model, these two operations are handled separately and thus result in inefficient terminal performance. The proposed algorithm unifies these processes in one framework that, based on simulated tests, reduces overall operating time by 15–20% in large vessels. This result indicates the possible application of hybrid approaches toward streamlining terminal operations for overall efficiency. The model in this research only addresses container handling tasks, with no indication as to scheduling of maintenance or other delays dependent upon interaction of the quay cranes with other equipment supporting such cranes. The authors indicate that in future research, the algorithm can be extended to include the maintenance and resource allocation considerations in the optimization framework. To tackle the issue of quay crane scheduling with uncertain handling times, Wang and his associates put forth a hybrid genetic algorithm ^19^. Their model GA showed efficiency in managing schedule robustness in varying terminal conditions, proving GAs can be useful for real world problems. But more automated terminal would have significant downtimes due to unplanned equipment failures or maintenance windows that Wang’s approach disregards. Zhao et al. introduced an improved GA for multi-objective quay crane scheduling, balancing efficiency, energy consumption, and resource utilization in automated terminals ^20^. While their method addresses multiple performance criteria, it does not consider the integrated planning of maintenance and job sequencing that our proposed model incorporates. In the same area, Chen and his colleagues addressed the problem of quay crane disruptions using a simulation-based genetic algorithm model ^21^. Their effort on dealing with responsive real-time breaks is very much in line with what we want to achieve. However, there is no thorough optimization framework that lacks a predefined integrated explorer which merges maintenance and operational activities, both scheduled and unscheduled. This is what we intend to work on.

Bukhsh developed an integrated model that jointly optimizes quay crane maintenance and scheduling using metaheuristic techniques ^22^. Their work represents an important step toward unifying downtime planning and task scheduling. Still, our model distinguishes itself by incorporating both planned and unplanned downtime scenarios in the context of automated terminals, with comparative analysis against multiple metaheuristics.

The article by Mekkaoui and Benabbou reviews the application of machine learning (ML) in port operations, particularly for predictive maintenance ^23^. It classifies ML applications into fault detection, failure prediction, and maintenance scheduling, highlighting their ability to analyze large datasets and detect patterns beyond traditional methods. The study emphasizes how ML models like neural networks and decision trees enhance equipment reliability and reduce unplanned downtime in container terminals. However, challenges remain in data availability, model interpretability, and real-time adaptability. The authors suggest integrating real-time sensor data and hybrid AI models to further improve predictive maintenance in automated terminals.

Thereafter, these authors mentioned that there has been lacking integration between a predictive maintenance model and real-time scheduling systems in the case of quay cranes and their supporting equipment. They believe that hybrid approaches, such as predictive analytics with dynamic scheduling for operational efficiency, are where future research should be directed. This study underlines the potential of ML in improving maintenance practices but also highlights significant gaps in integrating these techniques into holistic optimization frameworks.

The distribution of methodologies utilized in the reviewed literature (See Supplementary Table 1 and Supplementary Fig. 1) illustrates the dominant role of Genetic Algorithms and metaheuristic approaches, accounting for 50% of the reviewed studies. This reflects a clear trend in leveraging GA’s flexibility and scalability for complex quay crane scheduling and downtime reduction challenges. Other methodologies were each represented in smaller proportions, including:

- *Robust Optimization* (12%), which offers reliability in uncertain environments but suffers from high computational costs.
- *Mixed-Integer Programming* (12%), effective for maintenance scheduling but less scalable for real-time dynamic operations.
- *Machine Learning* (13%), showing promise for predictive maintenance though still limited by data integration and real-time responsiveness.
- *Systematic Review* (13%), useful for mapping current practices but lacking direct operational modeling.

This distribution highlights a shift in research focus toward metaheuristic and hybrid methods, while also revealing underexplored opportunities in integrated approaches particularly those that combine predictive analytics with adaptive scheduling, which this study aims to address.

The distribution of methodologies utilized in the reviewed literature is presented in Supplementary Table 1. This table summarizes key characteristics, such as the optimization method used, objective functions, and system types studied, offering context for our choice of a Genetic Algorithm-based approach.

Despite these advances, the existing literature still tends to address quay crane downtime in a fragmented manner, with most models focusing on either maintenance, scheduling efficiency, or operational disruptions in isolation. Recent studies have explored GA and hybrid metaheuristics for quay crane scheduling under integrated and low-carbon perspectives (Zheng ^24^, Eldemir & Taner ^25^). However, these approaches do not simultaneously capture planned downtime, unplanned failures, and idle inefficiencies within a unified framework. By integrating all categories of downtime within a single GA-based optimization model, this study directly addresses that gap and demonstrates comparative scalability in automated terminal contexts.

## Problem statement and mathematical formulation

Building on recent GA-based optimization studies in port and logistics operations, where metaheuristic approaches have demonstrated strong performance in solving multi-objective and constrained scheduling problems ^18,21,22,24,25^, this work advances the literature by explicitly modeling planned, unplanned, and idle downtimes within a single minimization-based objective function, thereby offering a more comprehensive representation of real operational conditions.

This study addresses the quay crane downtime problem in Tangier Med Port container terminals or in globally automated or semi-automated container terminals which need to be optimized. The goal here is to reduce the quay cranes’ idle or non-operational time, which has a detrimental impact on the terminal’s throughput and operational efficiency. Quay cranes are an important piece of equipment used in port operations. They move containers from the terminal to the ship. Due to planned or unplanned downtime, this causes delays in vessel servicing and raises operating expenses ^26^. Ineffective equipment coordination could potentially be the cause of this. Several types of handling equipment, including quay cranes, AGVs, straddle carriers, and ASCs, are included in a typical automated container terminal. These devices cooperate with one another to guarantee that the containers are carried through the terminal effectively. However, the entire procedure may be interrupted if a quay crane has some downtime. Two categories can be used to classify the type of downtime: planned and unplanned. To reduce the makespan of vessel handling operations and enhance the overall system performance, both must be kept to a minimum ^27^. As the link between container ships and the terminal yard, quay cranes (QC) play a crucial function ^28^. Meeting the necessary loading and unloading container schedule requires a QC to operate efficiently. However, QC downtime can be caused by a number of factors, including the following:

- *Planned Downtime*: This includes frequent maintenance, checks, software updates, or upgrades that are performed on the equipment ^29^. Even if the timing of the planned downtime can be arranged to minimize downtime, it still leads to the periods when a quay crane is not operating, and it still can be optimized more.
- *Unplanned Downtime*: This occurs due to unexpected technical failure, including mechanical, electrical, automation system failure ^30^, even up to the impact of weather conditions. The unplanned nature of this makes operations very much delayed because no one can expect it, and repairs or interventions take more time.

Additionally, quay cranes can also become idle when there is no vessel at berth to operate on or due to an inability of transfer equipment such as AGVs or straddle carriers to move containers. Quay cranes and other machinery cooperate to control the movement of containers at an automated terminal. These consist of:

*Automated Guided Vehicles*: They transit containers from quay to the yard or vice versa ^31^. These need to be integrated with the operations of the quay cranes so that congestion is avoided (See Supplementary Fig. 2).

*Automated Stacking Cranes (ASCs)*: The function of an ASC includes storing and retrieving of containers within the storage area ^31^. They must be used in parallel with AGVs or straddle carriers to facilitate seamless processing of the containers (See Supplementary Fig. 3).

The availability of AGVs, straddle carriers, and ASCs is crucial to the dock cranes’ operation cycle in container handling in order to maintain cycle continuity ^26^. Because the cranes must wait for the right equipment to be available for moving containers, any mismatch in the availability of these resources results in quay crane downtime. The many interdependent links between operations and equipment are the primary challenge in reducing quay crane downtime. Here are some examples of constraints:

*Resource Availability*: Availability of AGVs and straddle carriers at the quayside needs to be immediate once the quay crane finishes unloading a container. In case these vehicles arrive late, the resultant is crane idle time.

*Synchronization of Operations:* Co-ordination of quay cranes shall be optimum with other equipment in locale such as ASCs in the storage yard. Any unavailability of an ASC for receipt of containers may delay AGVs or a straddle carrier and thereby reducing crane availability.

*Planned Maintenance Windows*: These are supposed to be planned in such a way that they do not cause further disruptions, particularly when multiple vessels have to be serviced in the same timeframe.

*Weather and External Factors*: It includes factors that may be beyond one’s control, such as bad weather conditions-high winds, storms-which can completely bring the operation of the quay cranes to a standstill. These are not easy to predict, yet any optimization model would do well to include their consideration.

Our objective is to minimize the total downtime (D) of the quay cranes, which includes both planned downtime (D_S_) and unplanned downtime (D_US_), along with the idle time (D_id_) caused by operational inefficiencies. The mathematical model proposed for this optimization problem is as follows:

*Parameters (Input Data)*:

- Q: Set of quay cranes. (1)
- DP_q_: Planned downtime activities per crane (2)
- DP_qt_: Planned downtime duration per activity (3)
- DU_q_: Unplanned downtime activities per crane (4)
- DU_qt_: Unplanned downtime duration per activity (5)
- O: Other external factors (6)
- N: Set of Activities. (7)

*Decision Variables (Model Outputs)*:

- Iq: Total idle time per crane (8)
- DS: Total planned downtime (9)
- DUS: Total unplanned downtime (10)
- Did: Total idle downtime due to inefficiencies (11)
- D: Overall total downtime (12)

Where q ∈ Q.

The main objective is to minimize the total downtime D, which is a function of both planned and unplanned downtime, as well as idle time caused by inefficiencies, so the objective function can be written as follows:

13 $${\mathrm{Minimize}}\;\left( {\mathrm{D}} \right) = {\mathrm{DS}} + {\mathrm{DUS}} + {\mathrm{Did}}$$

where:

DS, represents the total planned downtime, including scheduled maintenance and other planned activities.

14 $${\mathrm{DS}} = \sum\limits_{n \in N} {{\mathrm{D}}_{{{\mathrm{Pqn}}}} *{\mathrm{D}}_{{{\mathrm{Pqnt}}}} } \quad {\mathrm{D}}_{{{\mathrm{Pqn}}}} \ge 0;{\mathrm{D}}_{{{\mathrm{Pqnt}}}} \ge 0$$

where D_Pqn_ ∈ DPq; and D_Pqnt_ ∈ DP_qt;_

DUS, represents the total unplanned downtime caused by unforeseen issues such as technical failures or weather conditions.

15 $${\mathrm{DUS}} = \sum\limits_{n \in N} {{\mathrm{DU}}_{{{\mathrm{qn}}}} *{\mathrm{DU}}_{{{\mathrm{qnt}}}} } \quad {\mathrm{DU}}_{{{\mathrm{qn}}}} \ge 0;{\mathrm{DU}}_{{{\mathrm{qnt}}}} \ge 0$$

where DU_qn_ ∈ DU_q_; and DU_qnt_ ∈ DU_qt_;

16 $${\mathrm{D}}_{{{\mathrm{id}}}} = {\mathrm{D}}_{{\text{idq }}} \quad {\mathrm{D}}_{{{\mathrm{idq}}}} \ge 0$$

represents the total idle time due to inefficiencies, such as waiting for AGVs, straddle carriers, or vessels.

This research seeks to minimize D, this means it will reduce planned downtime, DS, unplanned downtime, DUS, and idle time, Did. In practical terms, maximizing operational efficiency is achieved by minimizing the downtime components. Thus, the optimization model ensures reduced planned and unplanned disruptions, besides minimized idle time.

This formulation captures the critical factors that contribute to crane downtime. It accounts for both planned and unplanned disruptions, along with the inherent inefficiencies of the system that lead to crane idling. In addition, and in practice, unplanned downtime (DU_q_​) often follows a probabilistic distribution since it is caused by random failures or external conditions. Therefore, determining an accurate value for DU_q_ ​ requires incorporating reliability models or historical data on equipment failures, weather conditions, and other factors. Similarly, the idle time (D_id_) can be minimized by improving coordination between equipment and ensuring that resource allocation is optimized based on real-time operational data.

As an example, let consider the following values to calculate the Quay Crane Down Time:

- DPq = 2 planned maintenance activities, each lasting DPqt = 10 min,
- DUq = 1 unplanned maintenance activity, lasting DUqt = 15 min,
- Did = 20 min of idle time due to equipment unavailability and other external factors such as bad weather.

The total planned downtime (DS) as explained in (14) is the sum of the multiplication of DPq and DPqt but since we have only one crane for example then the formula will be as follow:

$${\mathrm{DS}} = {\mathrm{DPq}} \times {\mathrm{DPqt}} = 2 \times 10 = 20\;{\mathrm{minutes}}{.}$$

The total unplanned downtime (DUS) as explained in (15) is the sum of the multiplication of DUq and DUqt but since we have only one crane for example then the formula will be as follow:

$${\mathrm{DUS}} = {\mathrm{DUq}} \times {\mathrm{DUqt}} = 1 \times 15 = 15\;{\mathrm{minutes}}.$$

The idle time due to equipment unavailability and other factors as explained in (16) is equal to D_idq__=_ 20 for example.

The total downtime (D) is then calculated as:

$${\mathrm{D}} = {\mathrm{DS}} + {\mathrm{DUS}} + {\mathrm{Did}} = 20 + 15 + 20 = 55\;{\mathrm{minutes}}.$$

The total downtime in this example is 55 min, which illustrates the room for optimization. As D decreases, the value of Minimize(D) decreases, reflecting improved operational performance.

Although this model has not yet been deployed in live operations, it is designed around a weekly planning horizon. This frequency was selected in coordination with the operations team to allow regular review of maintenance needs, idle patterns, and breakdown events. A weekly cycle enables proactive adjustments and aligns with typical terminal maintenance planning practices.

## Proposed genetic algorithm approach

Unlike earlier GA-based approaches that focused only on either scheduling efficiency or disruption recovery, our model integrates planned, unplanned, and idle downtime categories within a single chromosome representation. This design not only reflects the complexity of real quay crane operations but also enhances the model’s practical relevance in automated terminal contexts.

**Fig. 1:**
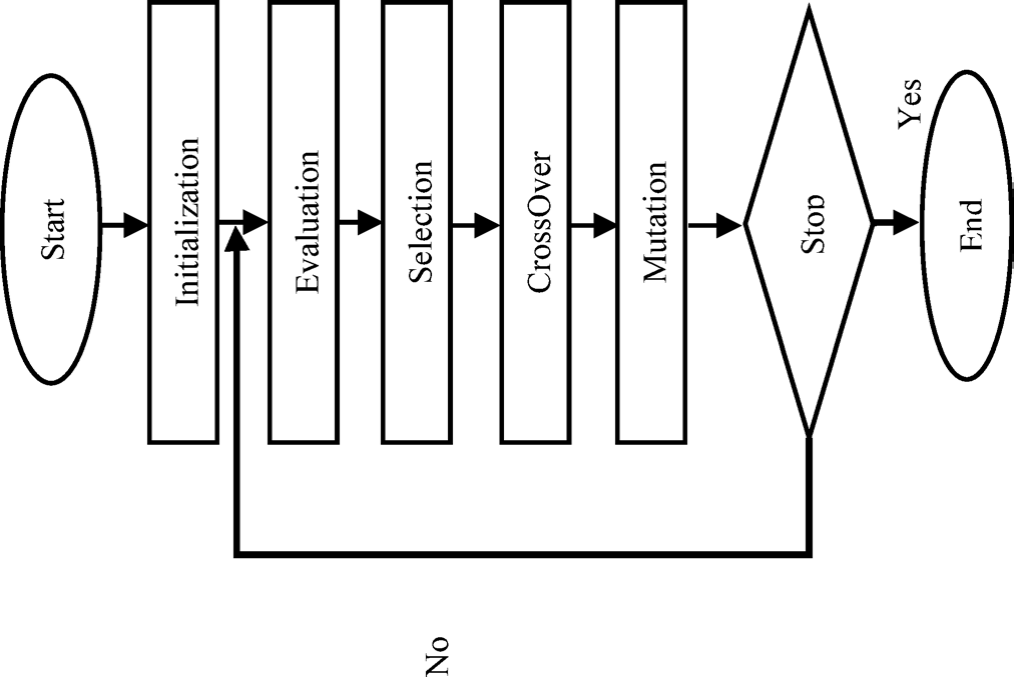
Genetic algorithm flow.

The process used to create an optimization model for the least amount of quay crane downtime is covered in great detail in this section. We employ GA, one of the most popular metaheuristic search techniques that simulates natural selection, to solve the downtime problem. By using operators like selection, crossover, and mutation to iteratively evolve a population of possible solutions (Fig. 1), it is very successful at resolving complicated optimization issues. In our concept, GA is used to look across a very wide search space for solutions that can reduce quay crane downtime, both planned and unplanned. Additionally, it looks for the best or almost best way to set up maintenance plans and the model’s operational efficiency.

Since the actual GA iteratively grows a population of potential solutions (Fig. 1), it is often regarded as being particularly capable of addressing challenging problems. One of the GA’s main advantages is its speedy coverage of a wide search space ^32^, which makes the algorithm better at locating local optima early in the optimization process. The algorithm’s quick convergence to a favorable area of the solution space offers a solid foundation for the GA’s potential to make significant gains early in the search. The GA operates by depicting potential solutions to the issue as chromosomes, as illustrated in Fig. 1; processes of selection, crossover, and mutation are involved ^33^.

**Fig. 2:**
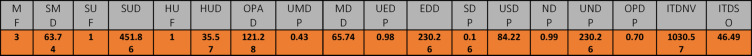
The proposed chromosome structure & example.

Our problem parameters are represented as a chromosome. Each chromosome is a vector containing key parameters related to quay crane maintenance and idle time (Fig. 2).

*Chromosome (Parameters)*

Each element in the chromosome (Fig. 2) corresponds to a specific operational parameter as detailed below:

- MF = Maintenance frequency
- SMD = Scheduled Maintenance Duration
- SUF = Software update frequency
- SUD = Software update duration
- HUF = Hardware upgrade frequency
- HUD = Hardware upgrade duration
- OPAD = Other planned activity duration
- UMDP = Unplanned mechanical downtime probability
- MDD = Mechanical downtime duration
- UEDP = Unplanned electrical downtime probability
- EDD = Electrical downtime duration
- SDP = Software downtime probability
- USDP = Unplanned Software downtime duration
- NDP = Network downtime probability
- UNDP = Unplanned Network downtime duration
- OPDP = Other unplanned downtime probability
- ITDNV = Idle Time due to No Vessel
- ITDSO = Idle Time due to Slow Operation

Chromosome = [D_S_, D_US_, D_id_]

The final chromosome includes parameters for software updates, hardware upgrades, and network-related disruptions in addition to other planned and unplanned parameters. These capture a more granular view of planned and unplanned downtime, enabling the model to address real-world complexities, such as overlapping activities or cascading failures.

*Planned Downtime (D*_*S*_ *)*

The planned downtime (DS) now accounts for all predictable activities, which can be scheduled in advance to reduce operational disruption:

(chromosome DS) DS = [MF, SMD, SUF, SUD, HUF, HUD, OPAD].

1 *Maintenance frequency*: Number of maintenance sessions per week or month.

2 *Scheduled maintenance duration*: Duration of each maintenance session.

3 *Software update frequency*: How often software updates occur.

4 *Software update duration*: Time required to perform each software update.

5 *Hardware upgrade frequency*: Frequency of hardware upgrades.

6 *Hardware upgrade duration*: Duration for each hardware upgrade.

7 *Other planned activity duration*: Time for inspections, audits, or other planned tasks.

*Unplanned Downtime (DUS)*:

The unplanned downtime (DUS) is now broken down into specific failure types, with individual probabilities and durations for each type of failure:

(chromosome DUS) DUS = [UMDP, MDD, UEDP, EDD, SDP, NDP, OPDP].

1 *Unplanned mechanical downtime probability*: Probability of mechanical failure.

2 *Mechanical downtime duration*: Average duration of mechanical downtime events.

3 *Unplanned electrical downtime probability*: Probability of electrical failure.

4 *Electrical downtime duration*: Average duration of electrical downtime events.

5 *Software downtime probability*: Probability of software-related failures.

6 *Software downtime duration*: Average duration of software downtime events.

7 *Network downtime probability*: Probability of network disruptions.

8 *Network downtime duration*: Average duration of Network downtime events.

9 *Other unplanned downtime probability*: Probability of other unforeseen failures.

*Idle Time (Did)*:

The idle time (Did) captures downtime caused by operational inefficiencies, such as waiting for vessels or slow operational cycles.

Did = [Idle time due to no vessel availability, Idle time due to slow operation].

*Idle time due to no vessel availability*: Time when cranes are idle because no vessels are docked or scheduled for loading/unloading.

*Idle time due to slow operation*: Time lost due to inefficiencies in the operation cycle, such as delays in equipment or staff.

With these parameters our functions will be like follow:

$${\mathrm{DS}} = \sum\limits_{n \in N} {{\mathrm{D}}_{{{\mathrm{Pqn}}}} *{\mathrm{D}}_{{{\mathrm{Pqnt}}}} } \quad {\mathrm{D}}_{{{\mathrm{Pqn}}}} \ge 0;{\mathrm{D}}_{{{\mathrm{Pqnt}}}} \ge 0;$$

where D_Pqn_ ∈ DPq; and D_Pqnt_ ∈ DP_qt_;

$${\mathrm{DS}} = {\mathrm{DP}} = \left( {\left( {{\mathrm{MF}}*{\mathrm{SMD}}} \right) + \left( {{\mathrm{SUF}}*{\mathrm{SUD}}} \right) + \left( {{\mathrm{HUF}}*{\mathrm{HUD}}} \right) + \left( {{\mathrm{OPAD}}} \right)} \right)$$

$${\mathrm{DUS}} = \sum\limits_{n \in N} {{\mathrm{DU}}_{{{\mathrm{qn}}}} *{\mathrm{D}}_{{{\mathrm{qnt}}}} } \quad {\mathrm{DU}}_{{{\mathrm{qn}}}} \ge 0;{\mathrm{DU}}_{{{\mathrm{qnt}}}} \ge 0;$$

where DU_qn_ ∈ DU_q_; and DU_qnt_ ∈ DU_qt_;

$$\begin{aligned} {\mathrm{DUS}} = {\mathrm{DUq}} = & \left( {\left( {{\mathrm{UMDP}}*{\mathrm{MDD}}} \right) + \left( {{\mathrm{UEDP}}*{\mathrm{EDD}}} \right)} \right. \\ & \left. { + \left( {{\mathrm{SDP}}*{\mathrm{USDP}}} \right) + \left( {{\mathrm{NDP}}*{\mathrm{UNDP}}} \right) + \left( {{\mathrm{OPDP}}} \right)} \right) \\ \end{aligned}$$

$${\mathrm{D}}_{{{\mathrm{id}}}} = {\mathrm{D}}_{{{\mathrm{idq}}}} \quad {\mathrm{D}}_{{{\mathrm{idq}}}} ;$$

In the other hand the model is designed to be applied to multiple cranes. For each crane, the total downtime (D) is calculated, and the goal is to minimize the aggregate downtime across all cranes:

$${\mathrm{Minimize}}\left( {{\mathrm{TD}}} \right) = \sum\limits_{n \in N} {{\mathrm{Minimize}}\left( {\mathrm{D}} \right)}$$

Quay crane operations are by nature tied to the availability and synchronization of other equipment at the terminal, such as AGVs and ASCs. AGVs move containers between quay cranes and storage yards, while ASCs handle the stacking and retrieval of containers (See Supplementary Fig. 4). Delays in either the availability of AGVs or the operations of ASCs will directly increase crane idle time, as the cranes must wait for the completion of container handoffs (See Supplementary Fig. 5). Crane idle times are not only due to maintenance activities but also, as explained before, to the complex interdependencies between quay cranes, AGVs, and ASCs. So, the delay came from AGV’s or ASC’s causing cranes to remain idle until the resources are available. This dynamic shows the intricate relationship between different terminal components and their collective impact on crane downtime. Idle time (IT) in our model is defined as delays due to unavailability of AGV and ASC in addition to some other root causes. For instance, if AGVs are held up in container pickup or drop, quay cranes cannot carry out unloading or loading and, thus, have to stay idle. In case the ASC is engaged in either stacking or retrieval operations, then cranes must wait, adding to idle time. Optimizing idle time thus indirectly corrects coordination inefficiencies. As an example, If an AGV/ASC takes an additional 10 min to transport a container due to congestion, the quay crane remains idle for that duration, increasing total idle time.

To ensure the model reflects realistic operational conditions (See Supplementary Table 2), and since we could not obtain real operational data from a container terminal, we simulated synthetic data that closely resembles real-world conditions. The parameters in this dataset are constrained within specific ranges. A detailed list of the GA model parameters and their operational ranges is provided in Supplementary Table 2. These constraints were selected based on convergence testing and reflect realistic limits applicable to quay crane maintenance scheduling.

$$\begin{aligned} {\mathrm{(A)}}\quad {\mathrm{Function}} & = \left\{ {\left( {\left( {{\mathrm{MF}}*{\mathrm{SMD}}} \right) + \left( {{\mathrm{SUF}}*{\mathrm{SUD}}} \right) + \left( {{\mathrm{HUF}}*{\mathrm{HUD}}} \right) + \left( {{\mathrm{OPAD}}} \right)} \right)} \right\} \\ & + \left\{ {\left( \begin{gathered} \left( {{\text{UMDP }}*{\text{ MDD}}} \right) + \left( {{\text{UEDP }}*{\text{ EDD}}} \right) \hfill \\ + \left( {{\text{SDP }}*{\text{ USDP}}} \right) + \left( {{\mathrm{NDP}}*{\mathrm{UNDP}}} \right) + \left( {{\mathrm{OPDP}}} \right) \hfill \\ \end{gathered} \right)} \right\} \\ & + {\mathrm{Did}} \\ \end{aligned}$$

**Fig. 3:**
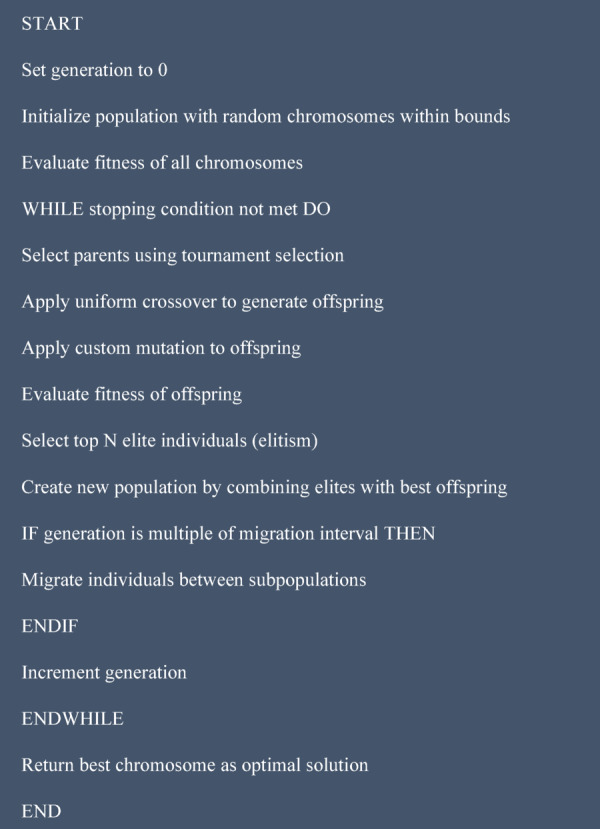
Genetic algorithm pseudocode.

**Fig. 4:**
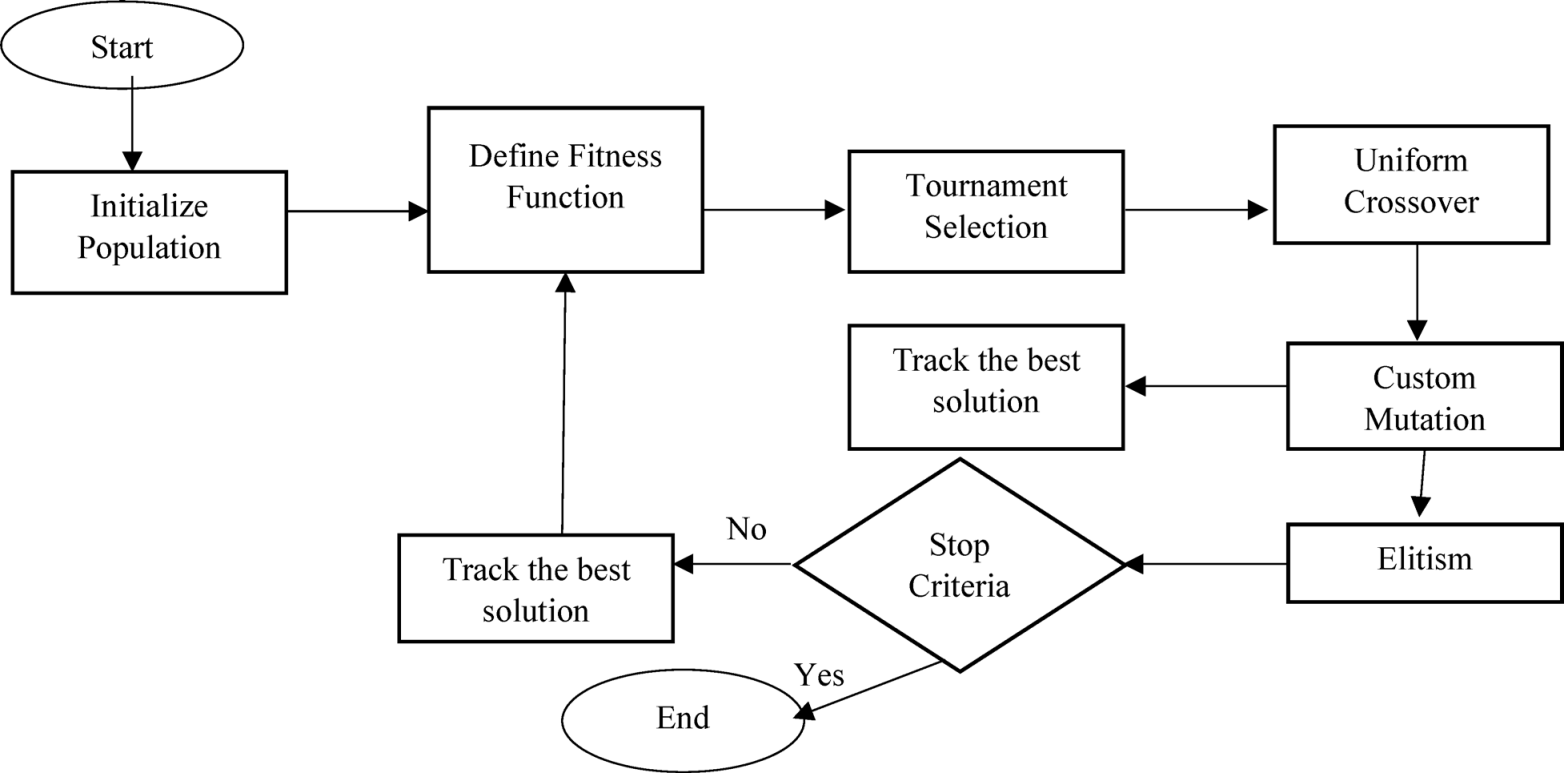
GA process.

In our approach (A) and as explained in the pseudocode (Fig. 3), we adopted a more robust set of Genetic Algorithm (GA) operators to address specific challenges identified in earlier iterations ^30^, particularly related to maintaining diversity, respecting variable bounds, and avoiding premature convergence. So as selection operator we implemented tournament selection (Fig. 3) ^33^ to introduce diversity by selecting a variety of chromosomes from each generation (Fig. 4). This method allows us to Randomly select a subset of the population (of size k) and retain the fittest individual within the subset for the next generation and to ensure that high-quality solutions propagate while still giving weaker chromosomes a chance, thus maintaining population diversity. For crossover, we chose uniform crossover (Fig. 3) ^34^, which provides new opportunities for genetic chromosomes that may have been lost or overlooked in earlier generations (Fig. 4). With uniform crossover each gene from the parent chromosomes has a 50% chance of being inherited by the offspring, ensuring a fair mix of both parents and this approach could create new gene combinations that may not have existed in previous generations, helping the algorithm explore new regions of the solution space.

For the mutation we went with custom function but initially, we tested random mutation ^34,35^, but it introduced issues where the mutated genes exceeded the allowable bounds (Fig. 4). For example, A gene with a probability bound between 0 and 1 could mutate to a value greater than 1 or less than 0, which is illogical solutions. And to solve this, we implemented a custom standard mutation function with 10% probability. This function ensures Mutated values stay within the specified bounds for each gene. And the mutation respects the data type and logical limits (probabilities between 0 and 1). We incorporated elitism to ensure that the best-performing solutions are not lost between generations ^36^. In each generation, the top individual (chromosome) is retained unchanged. This guarantees that the highest-quality solutions are preserved and carried over, even as new genetic genes are introduced. And finally, to prevent premature convergence, we used migration ^37^. Every 10 generations, individuals from one part of the population are swapped with others to introduce new genetic material. So, with migration it does shakes up the population, which reduces the risk of stagnation at local optima. And also helps maintain diversity by introducing new gene combinations from different subpopulations.

With this GA model, we conducted a parameter tuning process by systematically varying key parameters such as population size, number of generations, crossover type, and migration frequency to determine the optimal configuration of the Genetic Algorithm (GA).

Table 1 summarizes the parameter ranges tested and the selected values based on their impact on solution quality and convergence speed.

**Table 1:** Parameters tuning.

Parameter	Range Tested	Selected Value	Observations
Generations	100–1,000 (step: 100)	500	Increasing generations improved solution quality but had diminishing returns beyond 500
Population Size	20–100 (step: 10)	50	Lower population sizes led to premature convergence; larger sizes increased runtime without significant improvement
Mating	Uniform, Single-Point, Multi-Point	Uniform	Uniform crossover provided a balance between exploration and exploitation
Elitism	Top 1–Top 5 solutions retained	Top 2	Retaining the top 2 solutions ensured steady convergence without stagnation
Migration	Every 5–20 generations	Every 10	Migration every 10 generations-maintained diversity and avoided local optima

Table 2 in the Supplementary Table document presents a snapshot of the generated data used in our GA model. However as observed, in the generated dataset there are some illogical values in certain genes. For example, Hardware upgrade frequency is listed as 0 in column 4, yet the corresponding maintenance duration has a value of 83.6281. This presents an issue: How can there be time allocated for Hardware upgrade when no hardware upgrade sessions are scheduled? While such values appear illogical, we decided to keep them in the dataset for a specific reason. The logic behind this decision lies in how these values are handled within the fitness function.

**Table 2:** Analysis result.

Number of QCs	Fitness Value	Convergence Time (Generations)	Computational Time (Seconds)
1	142.22	500	3.71
5	56.37	500	87.20
10	67.15	500	183.33
20	65.55	500	385.02

In our fitness function, we ensure that these illogical values do not affect the outcome. Each gene’s value is multiplied by the corresponding related value (frequency with duration, probability with duration). As a result:

Maintenance = Maintenance Frequency × Maintenance Duration.

If frequency = 0, the contribution to the total downtime will automatically be 0, regardless of the duration value.

Supplementary Table 3 contains a representative sample of the synthetic dataset generated for GA training and testing. It includes input parameters such as downtime durations, probabilities, and idle time components used during evaluation of each chromosome’s fitness.

**Fig. 5:**
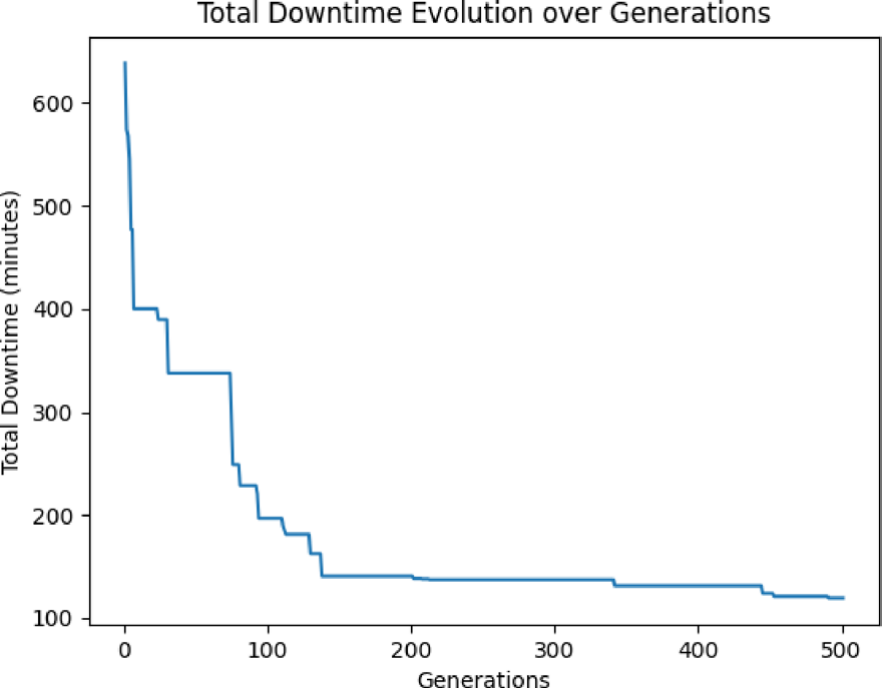
Final GA result.

The graph provided (Fig. 5) illustrates the evolution of total downtime over generations as the Genetic Algorithm (GA) was executed on the same generated dataset (Fig. 5). As observed from the plot, the algorithm demonstrates significant progress in minimizing total downtime, as an initial Phase (Generations 1–100), The total downtime decreases rapidly from over 600 min to around 160 min, showing that the algorithm quickly identifies promising solutions early on. Then on the second phase (Generations 130–330), The decline becomes more gradual as the GA refines the solutions and converges toward more optimized configurations. And in the final Phase (After Generation 450): The downtime stabilizes around 100 min (exactly 119.44min), indicating that the algorithm has likely found a near-optimal solution (Fig. 5). Minor fluctuations are observed, but the total downtime remains mostly constant from generation 450 onward. This graph demonstrates that the GA is functioning effectively, with steady convergence and no signs of premature stagnation. The combination of custom mutation, tournament selection, and uniform crossover ensures continuous exploration, while the elitism operator helps retain the best solutions across generations. By incorporating detailed parameters for planned and unplanned downtime, the model provides actionable insights for terminal operators. For instance, it can recommend optimal schedules for hardware upgrades to avoid peak operational times, minimizing disruptions to vessel servicing.

Compared with recent GA-based optimization studies addressing quay crane scheduling and terminal operations ^18,21,22,24,25^, the proposed GA formulation introduces a novel chromosome representation that explicitly encodes planned, unplanned, and idle downtimes within a unified objective function. This integrated encoding improves both interpretability and responsiveness to real operational conditions, while previous works typically optimized individual downtime components or assumed fixed maintenance intervals. Consequently, the present approach contributes to a more holistic and operationally relevant GA framework for container terminal optimization.

## Evaluation of scalability and sensitivity

**Fig. 6:**
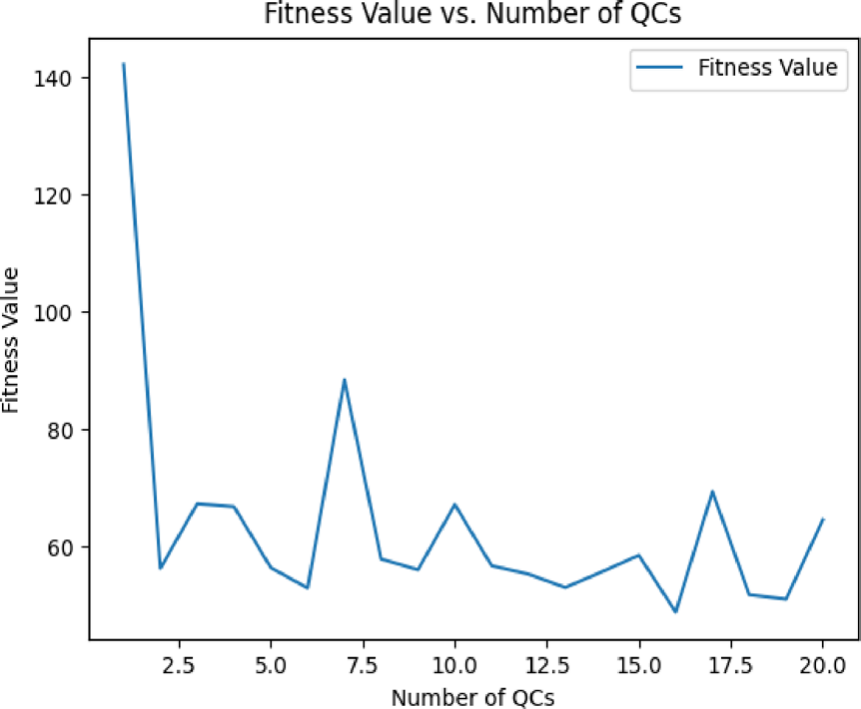
Fitness Value vs. Number of QCs.

**Fig. 7:**
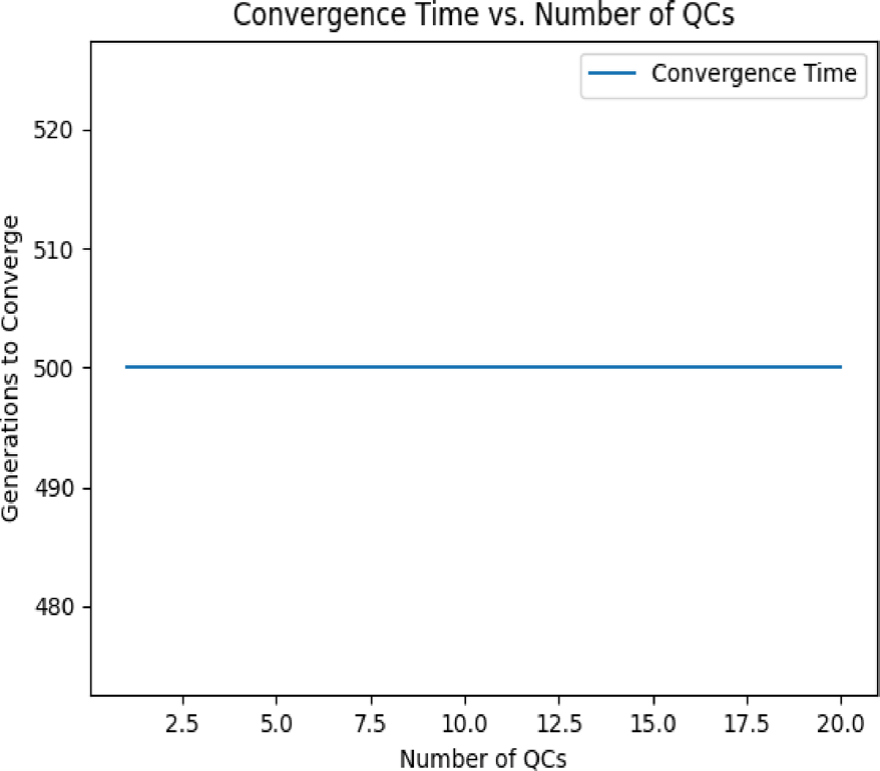
Convergence Time vs. Number of QCs.

**Fig. 8:**
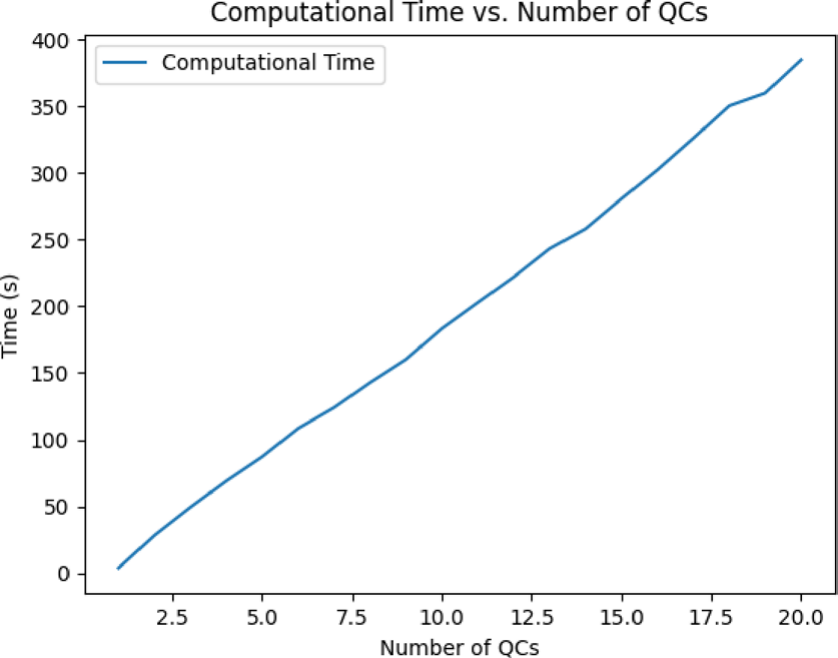
Computational Time vs. Number of QCs.

To validate the robustness and efficiency of our proposed Genetic Algorithm (GA), we conducted a Scalability and Sensitivity Analysis. This analysis was designed to assess the algorithm’s performance under varying problem sizes and input conditions. Specifically, we evaluated the scalability of the GA by progressively increasing the number of quay cranes (QCs) from 1 to 20. Key metrics, including fitness value (representing minimized quay crane downtime), convergence time (measured in generations), and computational time (total runtime in seconds), were recorded for each configuration. The results, summarized in (Table 3), indicate that the GA maintained stable fitness values across different problem sizes, demonstrating its robustness in handling increasing complexity. (Fig. 6) illustrates how the fitness value fluctuates slightly with the number of QCs, particularly for datasets with higher interdependencies (10–20 QCs). The convergence time, as shown in (Fig. 7), remained constant at 500 generations for all tests due to the predefined generation limit, suggesting that additional iterations may be required for larger datasets to achieve full convergence (Table 3). As expected, computational time, displayed in (Fig. 8), increased linearly with the number of QCs, reflecting the higher computational demands of larger problem sizes.

**Table 3:** Algorithms result comparison.

Algorithm	Avg. Final Downtime (min)	Avg. Convergence Gen	Stability	Exploration vs. Exploitation
GA	98.3	200	Moderate	Balanced
PSO	99.5	50	High	Strong exploitation
SA	197.8	470	Low	Strong exploration

These findings highlight the scalability of the GA while identifying areas for improvement. While the algorithm performs well for up to 20 QCs, further optimizations such as parallel processing or hybrid methods could improve computational efficiency for even larger datasets (Fig. 6). Additionally, increasing the generation limit for larger datasets could enhance solution quality by allowing more time for convergence.

## Performance analysis and comparative study

To improve that our GA model is applicable in this case we run our data and our config into two additional optimization algorithms: Particle Swarm Optimization (PSO) and Simulated Annealing (SA).

*Particle Swarm Optimization (PSO)*:

PSO is a population-based algorithm inspired by the social behavior of birds flocking or fish schooling. In PSO, each particle represents a potential solution, and particles move through the solution space influenced by their personal best-known position and the global best-known position found by the swarm.

In order to run the code on our data, we specified the following criteria’s:

*Population Size*: 100 particles, which allows a thorough exploration of the solution space.

*Generations*: 500 iterations, providing sufficient time for the particles to converge.

*Inertia Weight (w)*: Starting with a high inertia weight (0.9) to encourage exploration, gradually reduced over time.

*Cognitive and Social Coefficients (c1 and c2)*: Set to 1.5 each, balancing between the particle’s best position and the global best position.

**Fig. 9:**
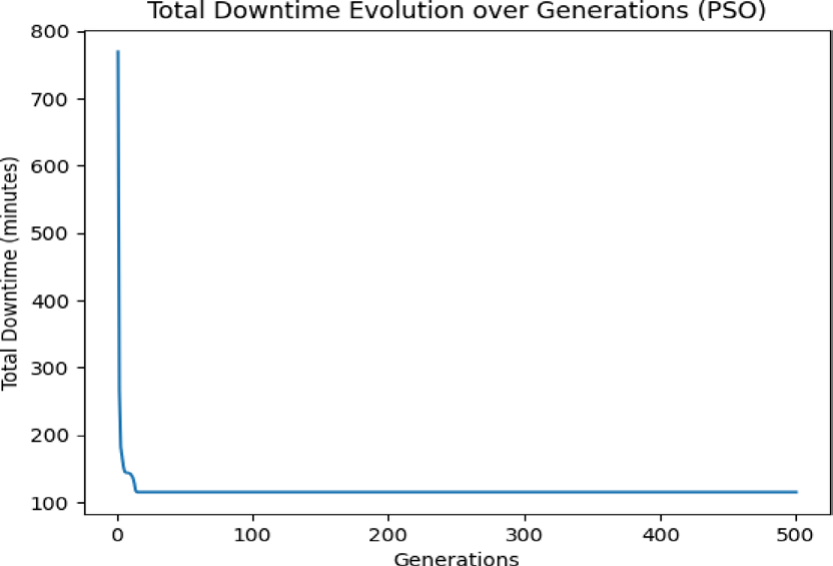
Final PSO result.

The PSO algorithm was able to reach a good solution quickly due to its high convergence rate (Fig. 9). This algorithm favors fast convergence by focusing on the global best-known solutions early in the optimization process.

*Simulated Annealing (SA)*

SA is a probabilistic algorithm inspired by the annealing process in metallurgy, where a material is heated and then slowly cooled to achieve a stable structure. In SA, the solution “temperature” gradually decreases, allowing the algorithm to accept worse solutions with decreasing probability as it progresses, which helps to escape local minima.

In order to run the code on our data, we specified the following criteria’s:

Initial Temperature: Set to 1000, allowing for significant exploration early on.

Cooling Rate: 0.98, meaning the temperature gradually decreases with each iteration, making the algorithm more selective in accepting worse solutions over time.

Generations: 500 iterations, allowing time for the solution to “cool” and stabilize near an optimum.

**Fig. 10:**
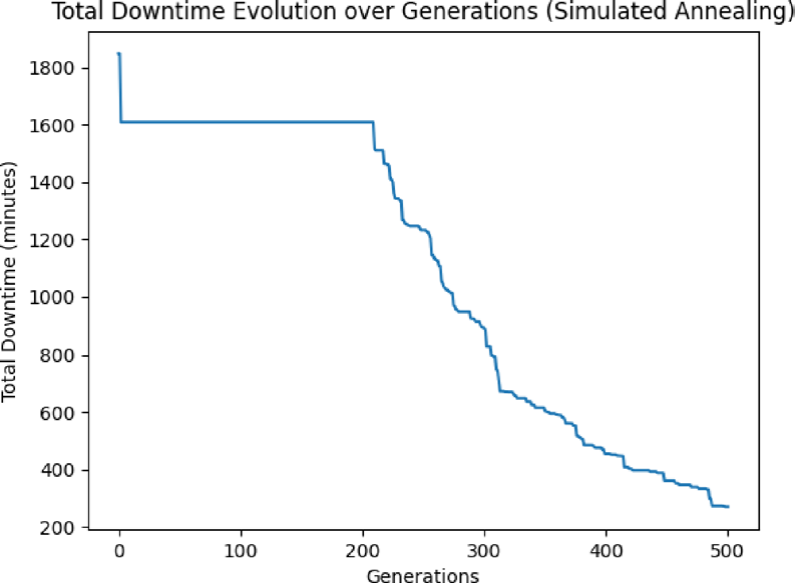
Final SA result.

SA offers a more balanced approach between exploration and exploitation, with a strong emphasis on escaping local optima. It explores broadly at the beginning, then refines solutions as the temperature decreases, gradually favoring more optimal configurations (Fig. 10).

*Convergence Speed*

*PSO*: Fastest convergence, with downtime stabilizing within the first 50 generations (Fig. 9). PSO’s rapid convergence is beneficial if a quick solution is needed.

*GA*: Slower than PSO but steadily improves the solution (Fig. 5), reaching convergence around generation 200.

*SA*: Slowest convergence, with downtime continuing to decrease gradually over the full 500 generations (Fig. 10). SA’s slower convergence indicates a thorough exploration but less immediate refinement.

*Final Downtime Value*

*PSO and GA*: Both reached similar minimum downtimes around 100 min (Figs. 5, 9), suggesting they were able to find optimal or near-optimal solutions.

*SA*: Reached a final downtime around 200 min (Fig. 10) within 500 generations. This is higher than the values achieved by PSO and GA, indicating that SA may need more generations or a slower cooling rate to reach comparable results.

*Stability*

*PSO*: Achieved a stable solution quickly after initial convergence, with minimal fluctuations.

*GA:* Showed some minor fluctuations after convergence, indicating ongoing refinements.

*SA:* Maintained more variation throughout, reflecting its ongoing exploration even at lower temperatures.

This configuration suggests that the GA favored moderate-to-high levels of preventive maintenance (Table 4), especially in response to components with higher failure probabilities, such as electrical systems. Idle times were significantly reduced, indicating effective coordination between quay cranes and support equipment like AGVs and ASCs. The algorithm balanced preventive interventions with realistic operational constraints, leading to improved total downtime. Due to the problem’s complexity and the lack of an exact analytical solution, theoretical optimal values are not available. However, the high consistency across repeated runs and the competitive performance relative to PSO and SA (Table 2) support the near-optimality of GA outcomes.

**Table 4:** Sample best-performing GA solution configuration.

Parameter	Value
Maintenance frequency	1 (per week)
Schedule maintenance duration	120 Minutes
Software update frequency	1 (per Month)
Mechanical failure probability	0.5
Electrical failure probability	0.4
Unplanned software downtime	45 Minutes
Idle Time (No Vessel)	240 Minutes
Idle Time (Slow Operation)	5 Minutes

As a conclusion PSO is faster at reaching a good solution and stabilizes quickly. If speed is a priority, PSO might be preferable. While both GA and PSO achieve similar final downtime values (~ 100 min), GA demonstrated a slightly lower average and better refinement behavior, as evidenced by its longer convergence and ongoing improvements past generation 200 (Table 2). In contrast, PSO converged faster but with less variability and adaptability. SA, although slower, proved useful for thorough exploration, particularly in problem spaces with complex local minima, but needs more iterations or tuning to match GA/PSO accuracy (Figs. 5, 9, 10).

## Conclusion

This research focused on the problem of reducing quay crane downtime at the automated container terminals, focusing on Tangier Med Port as case of study. For this purpose, a Genetic Algorithm (GA) was designed to optimize the maintenance scheduling in order to minimize both the idle time as well as unplanned downtime. The GA performed well because it was successful in balancing exploration and exploitation and was able to converge to high-quality solutions while avoiding premature convergence. Benchmarking evaluations against two other widely accepted metaheuristic algorithms, Particle Swarm Optimization (PSO) and Simulated Annealing (SA). While PSO was able to reach a solution faster than the rest, SA was the most adept at exploring the solution space to avoid local optima. GA was the best out of all three in overall balance, repeating the same optimized results but with different initial conditions. In particular, GA achieved the lowest average downtime (98.3 min), outperforming PSO (99.5 min) and SA (197.8 min), which confirms its superior effectiveness and scalability.

Although the findings demonstrate how effectively GA reduces crane downtime, the study also has several limitations. The first evaluation used simulated data along with the idealized operational scenarios which did not take into account real-life constraints such as weather, psychology of personnel involved, or scheduling that is subject to constant changes. The second model in turn did not allow for the use of real terminal data and real-time adaptive re-optimization, instead employing fixed input parameters. Combining GA with other algorithms (such as GA-RL or GA-NN) to exploit the complementary weaknesses of each other would create stronger improvement to this study. Also, validating the model using real-time operational data from actual container terminals would increase the practicality of the model. More advanced adjustment features or those that forecast periods of inactivity based on dynamically changing parameters and preceding data could make important contributions as well. All these approaches seem promising for the enhancement of intelligent optimization frameworks in maritime logistics and terminal operations. Furthermore, this study focused exclusively on automated container terminals, where cranes and vehicles (AGVs, ASCs) are governed by centralized software scheduling. Manned operations introduce additional uncertainty due to human intervention, reaction delays, and manual coordination. While these factors were not considered in the current model, the framework could be extended to accommodate manned terminal environments by introducing stochastic delays and behavioral variability. This represents a promising direction for future work, particularly in comparing automated versus manual crane handling strategies in terms of operational efficiency and resilience.

## Supplementary Information

Supplementary Information 1.

Supplementary Information 2.

Supplementary Information 3.

Supplementary Information 4.

## Data Availability

The datasets generated and analyzed during the current study are available in Google Drive: https://drive.google.com/drive/folders/1VG7nDxJQ91Us_7mdRDPGWmbmw4I8ss4E?usp=drive_link**.** Alternatively, the datasets used in this study are available from the corresponding author upon reasonable request.

## Abbreviations

PSO: Particle swarm optimization
SA: Simulated annealing
GA: Genetic algorithm
QC: Quay crane
ACT: Automated container terminals
AGV: Automated guided vehicles
ASC: Automated stacking cranes
ATLH: Automated twistlock handling
CRP: Container relocation problem
TL: Twistlock
ID: Idle time
DP: Planned downtime
DU: Unplanned downtime
FS: Maintenance frequency
TS: Scheduled maintenance duration
TU: Unscheduled maintenance duration
PS: Probability of unscheduled downtime
