# Supplementary material for: Minimizing quay crane downtime in container terminals using genetic algorithms with a case study of Tangier MED Port
Source: Sci Rep. 2025 Nov 23;15:45171. doi: 10.1038/s41598-025-29190-0 (PMC12749770; doi:10.1038/s41598-025-29190-0)
Supplement: Supplementary file 1 — Supplementary Information 1. [file 41598_2025_29190_MOESM1_ESM.docx]

**Supplementary Data File: Raw GA Input-Output**

This Excel file (Supplementary Data – Raw GA Input-Output) contains raw input-output trial data generated by the Genetic Algorithm for downtime minimization at Tangier MED Port.

These data were used to support the analysis shown in Supplementary Table 3.
