# Supplementary material for: Minimizing quay crane downtime in container terminals using genetic algorithms with a case study of Tangier MED Port
Source: Sci Rep. 2025 Nov 23;15:45171. doi: 10.1038/s41598-025-29190-0 (PMC12749770; doi:10.1038/s41598-025-29190-0)
Supplement: Supplementary file 2 — Supplementary Information 2. [file 41598_2025_29190_MOESM2_ESM.docx]

# Supplementary Tables

## Supplementary Table 1. Summary of Related Works

Legend: This table compares prior research related to maintenance and optimization in port operations, including methods used, key findings, and identified research gaps.

| Article | Focus | Methodology | Key Findings | Gaps Identified |
| --- | --- | --- | --- | --- |
| Li and Hu [17] | Preventive maintenance scheduling | Mixed-integer linear programming | Optimized intervals, 15% downtime reduction | No real-time adaptability |
| Đurasević and Đumić [15] | Container relocation optimization | Genetic programming | 4–6% improvement in efficiency | Limited to yard operations |
| Wang et al. [16] | Berth and crane scheduling | Robust optimization | Improved reliability under uncertainty. | No maintenance integration |
| Rahman et al. [18] | Container handling optimization | Hybrid genetic algorithm | 15–20% reduction in operation time | No maintenance or interdependency consideration |
| Chen et al. [21] | Responsive crane disruptions | Simulation-based GA | Managed real-time disruptions | No proactive scheduling |
| Zhao et al. [20] | Multi-objective scheduling | Improved GA | Balanced efficiency, energy use | No integrated downtime model |
| Bukhsh et al. [22] | Integrated maintenance + scheduling | Metaheuristic model | Unified scheduling + maintenance | No unplanned downtime coverage |
| Mekkaoui et al. [23] | ML in port predictive maintenance | Systematic review | Improved failure prediction and scheduling | Challenges in data integration, limited real-time adaptability |

## Supplementary Table 2. GA Model Parameter Ranges

Legend: Describes the operational ranges and constraints used for GA modeling of quay crane maintenance planning.

| Variable | Range | Notes |
| --- | --- | --- |
| Maintenance frequency | 0 to 3 sessions per week | Based on operational needs |
| Scheduled maintenance duration | 5 minutes to 4 hours | Varies per session |
| Software update frequency | 0 to 2 updates per week | Regular updates |
| Software update duration | 30 minutes to 8 hours | Includes major updates |
| Hardware upgrade frequency | 0 to 2 upgrades per week | Predictable upgrades |
| Hardware upgrade duration | 30 minutes to 8 hours | Time for upgrades |
| Other planned activity duration | 30 minutes to 8 hours | Inspections, audits, etc. |
| Unplanned mechanical downtime probability | 0 to 1 | Requires probabilistic model |
| Mechanical downtime duration | 30 minutes to 4 hours | Historical data required |
| Unplanned electrical downtime probability | 0 to 1 | Requires probabilistic model |
| Electrical downtime duration | 30 minutes to 4 hours | Historical data required |
| Software downtime probability | 0 to 1 | Requires probabilistic model |
| Unplanned Software downtime duration | 30 minutes to 4 hours | Historical data required |
| Network downtime probability | 0 to 1 | Requires probabilistic model |
| Unplanned Network downtime duration | 30 minutes to 2 hours | Historical data required |
| Other unplanned downtime probability | 0 to 1 | Requires probabilistic model |
| Idle time due to no vessel availability | 10 minutes to 48 hours | Operational inefficiency |
| Idle time due to slow operation | 5 minutes to 1 hour | Delays in processes |

## Supplementary Table 3. GA Input-Output Data for Sample Maintenance Scheduling Trial

Legend: Detailed configuration of maintenance frequencies, downtimes, and idle durations for a sample trial generated by the Genetic Algorithm during simulation.

| Maintenance Frequency (sessions/week) | 3 | 1 | 2 | 2 | 2 | 3 |
| --- | --- | --- | --- | --- | --- | --- |
| Scheduled Maintenance Duration (minutes) | 63.74198795 | 124.1715947 | 54.362849 | 54.9340829 | 89.3835184 | 205.2624025 |
| Software Update Frequency | 1 | 1 | 1 | 2 | 2 | 2 |
| Software Update Duration (minutes) | 451.86878602 | 151.928953 | 269.791600 | 352.535738 | 105.697085 | 385.0264059 |
| Hardware Upgrade Frequency | 1 | 2 | 2 | 0 | 1 | 2 |
| Hardware Upgrade Duration (minutes) | 35.5770732 | 352.15617 | 224.1773905 | 83.6281198 | 64.1989618 | 201.26761 |
| Other Planned Activity Duration (minutes) | 121.28791 | 415.593985 | 286.819460 | 471.0232671 | 469.2919921 | 304.964900 |
| Unplanned Mechanical Downtime Probability | 0.4382400 | 0.29541 | 0.741520 | 0.25367675 | 0.37716747 | 0.0545927 |
| Mechanical Downtime Duration (minutes) | 65.7414 | 144.3455 | 198.79988 | 64.844806 | 153.9361 | 153.984773 |
| Unplanned Electrical Downtime Probability | 0.980722 | 0.7448564 | 0.1411554 | 0.41473452 | 0.71415494 | 0.0133219158 |
| Electrical Downtime Duration (minutes) | 230.2687 | 130.497 | 119.93904 | 45.114390 | 157.2279 | 181.1910799 |
| Software Downtime Probability | 0.163983 | 0.1093435 | 0.2780204 | 0.7866669 | 0.022234629 | 0.608502006 |
| Unplanned Software Downtime Duration | 84.22173 | 45.22756 | 154.2423 | 93.883536 | 131.92847 | 221.68930 |
| Network Downtime Probability | 0.996759 | 0.35812 | 0.92018 | 0.89221 | 0.5904761 | 0.58541 |
| Unplanned Network Downtime Duration (minutes) | 230.2687 | 130.4971 | 119.939 | 45.114390 | 157.22796 | 181.19107 |
| Other Unplanned Downtime Probability | 0.70345 | 0.87093 | 0.88504 | 0.281175 | 0.550248 | 0.320226 |
| Idle Time due to No Vessel (minutes) | 1030.57 | 1585.82 | 1950.94 | 514.130289 | 204.99362 | 2185.989 |
| Idle Time due to Slow Operation (minutes) | 46.4976 | 39.335 | 47.49776 | 23.370826 | 10.349175 | 12.494887 |
