# Supplementary material for: Minimizing quay crane downtime in container terminals using genetic algorithms with a case study of Tangier MED Port
Source: Sci Rep. 2025 Nov 23;15:45171. doi: 10.1038/s41598-025-29190-0 (PMC12749770; doi:10.1038/s41598-025-29190-0)
Supplement: Supplementary file 4 — Supplementary Information 4. [file 41598_2025_29190_MOESM4_ESM.docx]

# Supplementary Figures

## Supplementary Figure 1. Methodologies Utilization

Legend: Visual representation of methodologies used in the reviewed literature, highlighting the dominance of metaheuristic approaches.

## Supplementary Figure 2. Automated Guided Vehicles (AGVs)

Legend: Illustration of AGVs used to transport containers between the quay and yard in automated terminals.


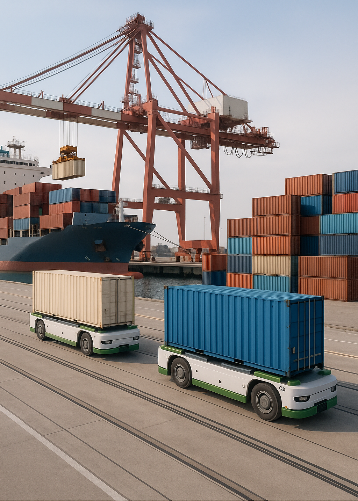


## Supplementary Figure 3. Automated Stacking Cranes (ASCs)

Legend: Example of ASCs used in conjunction with AGVs for efficient yard operations.


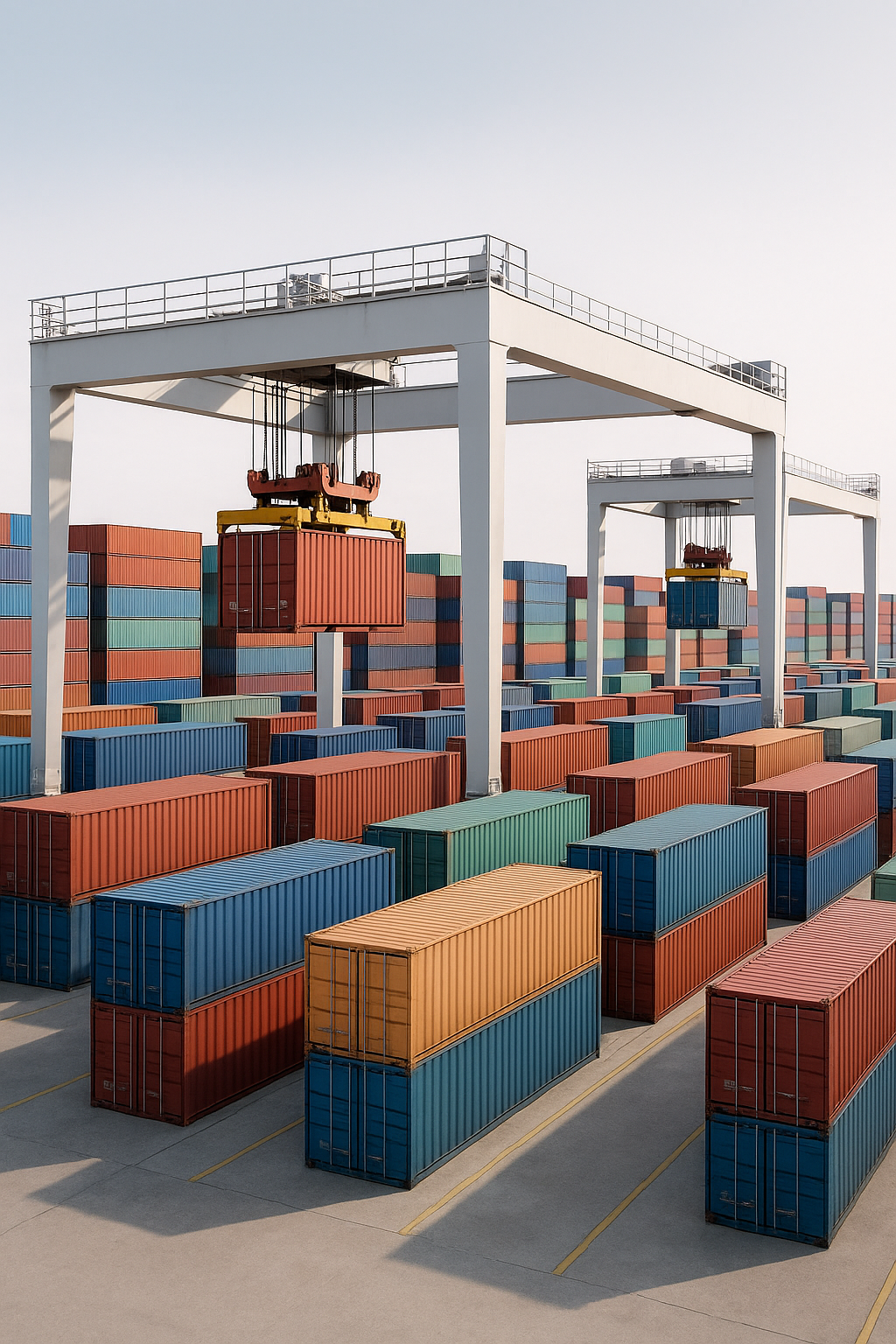


## Supplementary Figure 4. Terminal Structure Overview

Legend: Layout showing the typical arrangement of terminal areas and major equipment.


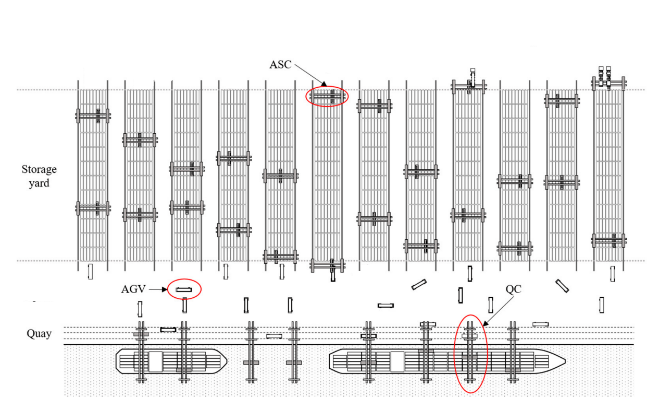


## Supplementary Figure 5. Equipment Relationship at Container Terminal

Legend: Diagram showing the interrelations between key port handling equipment including QCs, AGVs, and ASCs.

**Quay Crane** (Idle Time).


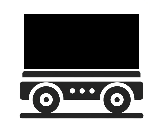

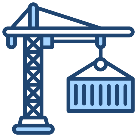

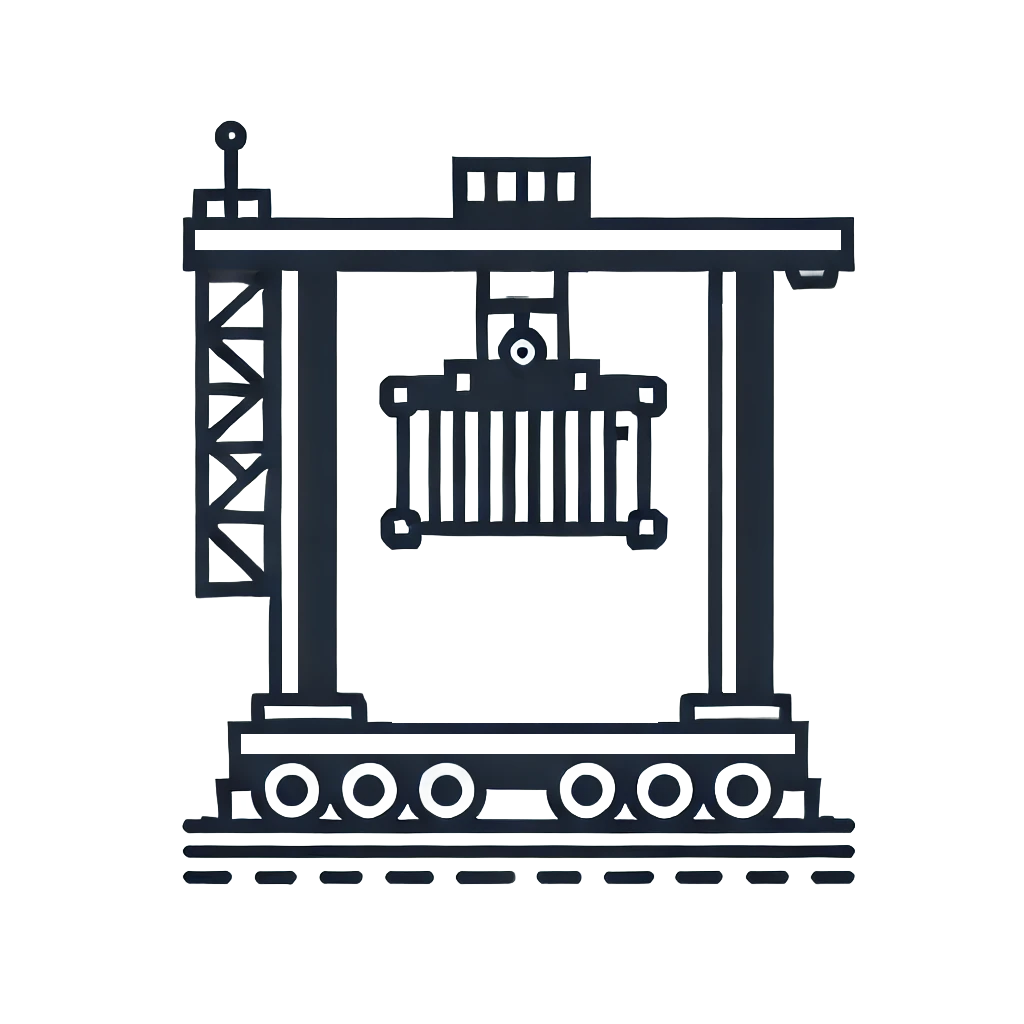


**AGV Delay**

**ASC Delay**

**Operation Delay**
